# Second Re-irradiation: Clinical Examples of Worthwhile Treatment

**DOI:** 10.7759/cureus.2813

**Published:** 2018-06-15

**Authors:** Carsten Nieder, Rosalba Yobuta, Bård Mannsåker

**Affiliations:** 1 Oncology and Palliative Medicine, Nordland Hospital Trust, Bodø, NOR; 2 Oncology, Nordland Hospital Trust, Bodø, NOR

**Keywords:** cancer, palliative radiotherapy, bone metastases, re-irradiation, toxicity

## Abstract

Introduction: Improved treatment approaches have resulted in longer survival of patients with certain types of incurable cancer, without eliminating the need for symptom palliation and supportive measures. In this context, re-irradiation is an increasingly important option. Little data exists about a second or repeat re-irradiation.

Methods: From a single institution database, patients who received a second re-irradiation with cumulative equivalent doses (equivalent dose in 2-Gy fractions (EQD2) for late effects, alpha/beta-value 3 Gy) of more than 90 Gy and survived for more than six months were identified. Illustrative clinical examples were provided.

Results: The examples describe the treatment of sacral bone metastases, recurrent rectal cancer, and pelvic lymph node metastases. The maximum cumulative EQD2 was 142 Gy. Symptomatic responses were obtained without clinically relevant side effects.

Conclusion: These three cases illustrate that a second re-irradiation has the potential to provide worthwhile palliative effects without causing overt late toxicity during the remaining life time. In patients who tolerated previous radiotherapy well, further re-irradiation may contribute to the ever-increasing armamentarium of options that increase the survival of patients with incurable cancer and try to prolong the time period where independent living is possible.

## Introduction

Improved treatment approaches have resulted in longer survival of patients with certain types of incurable cancer, without eliminating the need for symptom palliation and supportive measures [1-2]. In this context, re-irradiation is an increasingly important option [3-11]. Especially for painful bone metastases, strong evidence from a large phase III study suggests that re-irradiation often is worthwhile, even in situations where the first course did not result in satisfactory pain relief [12]. Given that symptomatic improvement, e.g., of bone pain, often is transient, several radiation oncology providers have started to offer a second re-irradiation to selected patients [13]. A recent review of the available evidence identified a limited number of studies, all with retrospective design [14]. Despite several limitations, the published data suggest the feasibility and safety of repeated re-irradiation for several, but not all anatomic locations. Relatively high rates of toxicity were described after cranial and thoracic treatments. Abusaris et al. have suggested a strategy to estimate normal tissue tolerance in this setting [15]. For critical organs at risk (OAR), the maximum dose was set as 50% more than the normal constraint, if the elapsed time interval was ≥12 months after the last radiation (recovery from occult damage). After six to twelve months, 25% more than the normal constraint was accepted. Prospective validation of this strategy is still pending. Clearly, more clinical data is needed to determine the appropriateness of a second re-irradiation. Therefore, we decided to provide illustrative examples, which may support decision-making and patient selection.

## Materials and methods

We have systematically collected treatment information for all re-irradiated patients since our radiation oncology facility was opened 10 years ago. Previously, the palliatively treated patients were analyzed in order to develop a prognostic model predicting survival [16]. For the present retrospective analysis, we identified patients who received a second re-irradiation to the same, previously treated volumes. Since the vast majority of patients were found to have received low cumulative total doses to non-spine bone metastases and had a survival of less than six months, i.e., insufficient follow-up to determine the long-term safety, we decided to focus on selected cases with longer follow-up and higher doses; patients in whom we are confident that treatment was worthwhile. Each of these teaching cases provides useful information for other clinicians managing previously irradiated patients. They complement a recently published scenario of repeat spinal re-irradiation [13]. We have not identified patients who suffered serious complications from a second re-irradiation in our electronic database. As a retrospective analysis of routine clinical care, no approval from the Regional Committee for Medical and Health Research Ethics (REK Nord) was necessary. Similarly, no approval from the Norwegian Social Science Database (NSD) had to be obtained. We calculated the biologically equivalent dose in 2-Gy fractions (EQD2) for late effects according to the linear-quadratic model with an alpha/beta-value of 3 Gy [17], without accounting for hyperfractionation. We followed the methods described by Abusaris et al. to estimate the cumulative total dose from all three courses after accounting for time-dependent recovery [15]. The treatment planning system was Varian Eclipse (Varian Medical Systems, Inc., Palo Alto, CA, USA). If possible, the images and treatment courses were co-registered to obtain cumulative dose distributions.

## Results

Case 1

**Figure 1:**
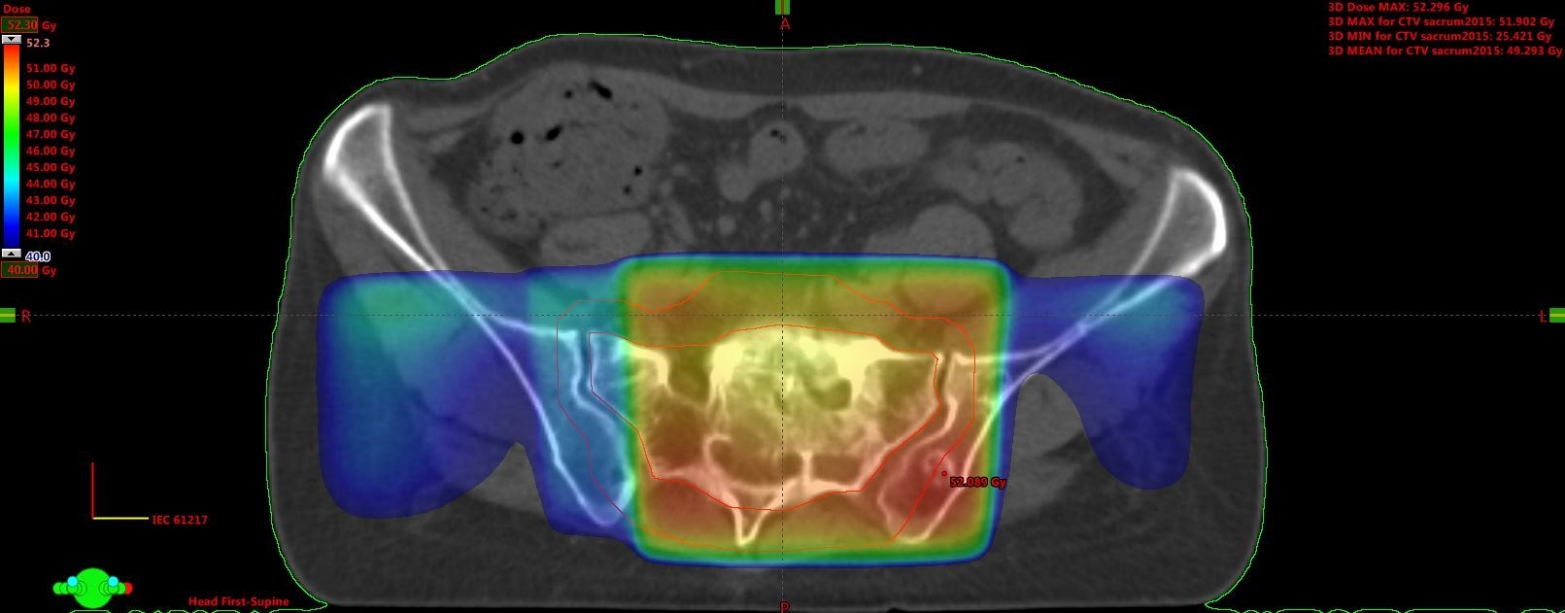
Dose distribution from the co-registered second and third course combined Axial computed tomography (CT) scan, doses >40 Gy are displayed, the maximum dose was 52 Gy. The bowels are located outside the 40-Gy isodose.

This female patient with a history of stage II breast cancer, treated in 1997, presented with painful sacral bone metastases in June 2011. She had biopsy-verified estrogen receptor positive HER-2 negative disease, both in the liver and bones, and was 60 years old. Several lines of sequential chemotherapies, endocrine treatments, and bone-targeted agents were employed from June 2011 onwards. Her Karnofsky performance status (KPS) was 90%. She received palliative radiotherapy to the sacral metastases, 10 fractions of 3 Gy, via a simple single posterior field. After an initial clinical improvement, the symptoms worsened in February 2012 and therefore, re-irradiation was offered. The same fractionation regimen was used; however, this time a 3-D conformal 3-field plan was employed. She responded again clinically and was later referred for the second re-irradiation in January 2015. At that time, her KPS was still 90%. The same 3-D conformal technique was used; however, only 8 fractions of 2.5 Gy were delivered. At the last clinical follow-up in November 2017, she received systemic therapy with pegylated liposomal doxorubicine. Each of the three courses was well tolerated and so far no chronic side effects have been observed. Figure 1 shows the cumulative radiation dose distributions from course two and three. No planning computed tomography (CT) had been performed for the first course.

All three courses combined resulted in an EQD2 for late effects of 94 Gy. When using the Abusaris et al. recovery terms [15], we have to subtract 25% from the initial EQD2 (time interval 6-12 months) and 50% from the second EQD2 (time interval ≥12 months). The resulting cumulative EQD2 for late effects from all three courses is as low as 67 Gy in this model.

Case 2

**Figure 2:**
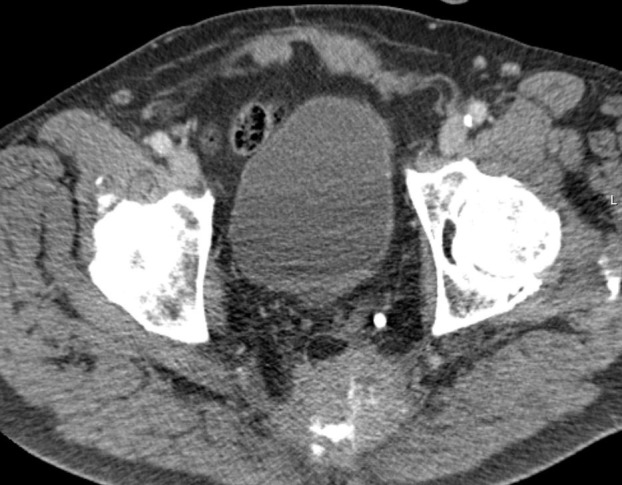
Axial computed tomography (CT) scan Large sacral and pre-sacral mass.

This 79-year-old male patient was initially irradiated in June 2008 when he was diagnosed with recurrent T4 N0 M0 rectal cancer after previous surgical resection (no (neo-)adjuvant therapy, abdominoperineal resection), Figure 2.

**Figure 3:**
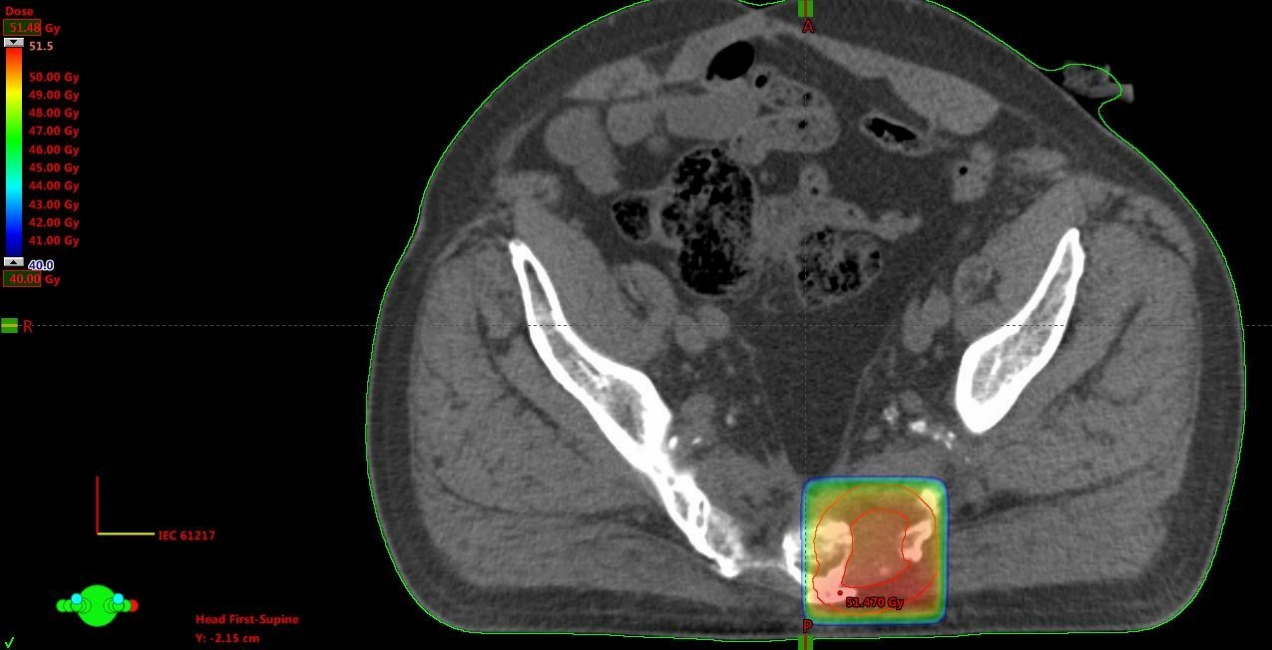
Axial computed tomography (CT) scan and dose distribution from the second course Doses above 40 Gy are displayed, maximum dose 51 Gy.

A 3-D conformal plan was employed (30 fractions of 2 Gy, concomitant capecitabine 825 mg/m2 twice daily). He experienced excellent pain relief and refused surgical resection. Due to increasing pain, he received re-irradiation in July 2009 (3-D conformal, 28 fractions of 1.8 Gy, concomitant capecitabine as detailed above) (Figure 3).

He was still free from nodal and distant metastases and had a KPS of 90%. Pain relief was excellent again. The final course of radiotherapy for palliation of pain was given in January 2012 (3-D conformal, 12 fractions of 2.5 Gy). At that time, untreated lung metastases were present, too. The patient’s KPS was 70%. Pain relief was incomplete and lasted for four months, during which reduced doses of analgesics were sufficient. The patient died in October 2013 after having received hospice care and advanced pain management. Each of the three courses was well tolerated. Due to sacral tumor infiltration, neurologic deficits developed gradually. Even if radiation-induced contributions are hard to judge, we did not observe any clear treatment-related late toxicity. All three courses combined resulted in an EQD2 for late effects of 142 Gy. When using the Abusaris et al. recovery terms [15], we have to subtract 50% from the initial EQD2 and from the second EQD2 (time interval ≥12 months). The resulting cumulative EQD2 for late effects from all three courses is 87 Gy in this model.

Case 3

**Figure 4:**
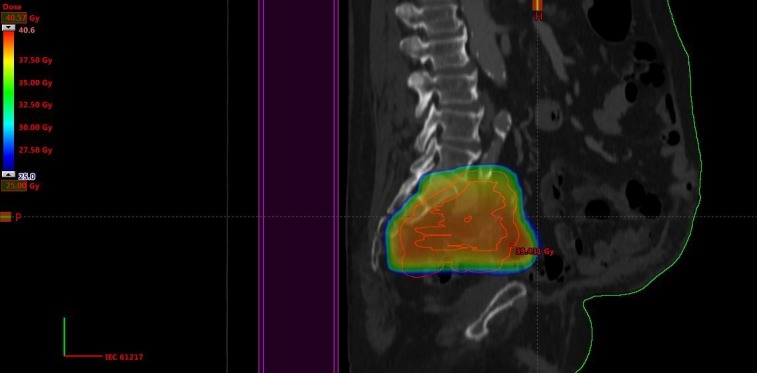
Sagittal computed tomography (CT) scan and dose distribution Doses above 25 Gy are displayed, maximum dose 41 Gy.

This 72-year-old male patient was initially irradiated in 2006 when he was diagnosed with prostate cancer (stage T3a, serum prostate-specific antigen (PSA) 12 ng/ml, Gleason score 3+3). A 3-D conformal plan was employed (38 fractions of 2 Gy, together with endocrine treatment). In January 2014, he was diagnosed with urothelial bladder cancer (stage T3a, grade 3) and cystoprostatectomy was performed (no (neo-)adjuvant therapy). A bilateral pelvic node relapse was diagnosed in January 2016. After discussion in the multidisciplinary tumor board, he received palliative radiotherapy with minimal dose overlap in the caudal, prostate-near region (13 fractions of 3 Gy, 3-D conformal plan, KPS 80%) (Figure 4).

**Figure 5:**
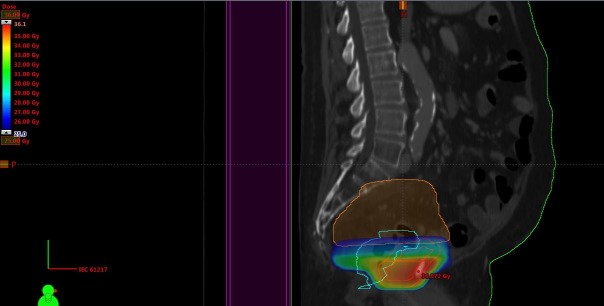
Sagittal computed tomography (CT) scan and dose distribution (course 3) Doses above 25 Gy are displayed, maximum dose 36 Gy.

Pain and lower extremity edema relief was obtained. In May 2016, progression occurred in additional node regions and we prescribed hyperfractionated radiotherapy, because of the short time interval and overlapping target volumes, this time at the caudal border of the previous nodal target volume, i.e., in the former prostate region where we initially had tried to reduce the volume of overlap (two daily fractions of 1.1 Gy, interval at least six hours, total dose 26.4 Gy with an additional simultaneous integrated boost of 0.3 Gy twice daily (7.2 Gy) to the nodes that had not been included in the January target volume (Figure 5). The main goal was pain relief.

**Figure 6:**
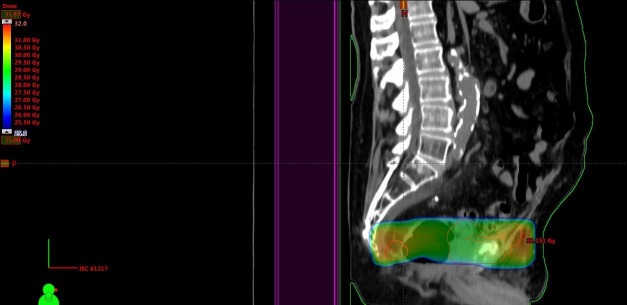
Sagittal computed tomography (CT) scan and dose distribution (course 4) Doses above 25 Gy are displayed, maximum dose 32 Gy.

The patient responded well, both clinically and radiologically. He was re-irradiated again in May 2017 to the former prostate region because of pain and radiological progression, at a time when his KPS had declined to 60% (Figure 6).

This time, two daily fractions of 1.5 Gy were employed; a total dose of 30 Gy. Pain improved until November 2017, when opioid treatment via pump became necessary. The patient deceased in December. Each of the courses was well tolerated. The exact cumulative total dose from all courses could not be determined, because of the substantial anatomy changes induced by cystoprostatectomy and the fact that we could not import the prostate treatment course from the now outdated planning system that was used in 2006. However, it is clear from Figures 4-6 that the former prostate region was included again in course three and four, resulting in at least 76 + 33.6 + 30 Gy (EQD2 for late effects at least 133 Gy). When using the Abusaris et al. recovery terms [15], we have to subtract 50% from the initial EQD2 and from the EQD2 of course three (time interval ≥12 months). The resulting cumulative EQD2 for late effects from all three courses is 80 Gy in this model.

## Discussion

These three cases illustrate that a second re-irradiation has the potential to provide worthwhile palliative effects without causing overt, clinically relevant late toxicity. Survival from the final radiotherapy course was more than 2.5 years (ongoing), 21 months, and seven months, respectively. With longer follow-up, late toxicity might still become clinically apparent. However, in many clinical situations, survival is too short to develop relevant late toxicity [16]. Hyperfractionation leads to lower EQD2 and might thus also contribute to a limited risk of side effects [13]. Unfortunately, prospective head to head comparisons of different fractionation concepts are not available in this setting. Nevertheless, data from the literature suggest that hyperfractionation should be considered for locally recurrent rectal cancer [18-19] and possibly also nasopharyngeal cancer [20-21]. Besides fractionation, highly conformal techniques, including brachytherapy, or in some cases proton beam irradiation may be able to reduce the volume of normal tissues exposed to high cumulative total doses [5,9,22-24]. As illustrated in Case three, the common policy of avoiding elective nodal irradiation in the re-irradiation setting sometimes results in relapses in adjacent nodes, which might be the source of new problems with overlapping target volumes and isodoses.

The individual concepts chosen to treat these three patients are debatable. Other strategies might have been chosen by other health care providers and in other regions of the world. For example, the breast cancer bone metastases (Case 1) may also have responded to short-course radiotherapy [25]. The 2.5-Gy x 8 bone re-irradiation regimen has also been used in the large phase 3 study that compared 8-Gy single fractions to a total dose of 20 Gy [12]. The bladder cancer patient (Case 3) may be eligible for an immune checkpoint inhibitor in the present era [26]. He was judged unfit for chemotherapy while we provided the radiotherapy courses described earlier. Clearly, a multidisciplinary discussion is recommended in these complex scenarios. Even if this small experience from three patients is not suitable to validate the Abusaris et al. recovery terms [15], our observations are compatible with the underlying hypothesis of time-dependent recovery, allowing for the administration of a cumulative EQD2 in excess of 120 Gy for late effects (alpha/beta-value 3 Gy), at least to limited volumes. Such strategies should only be pursued in patients who tolerated the previous radiotherapy well. If the time interval is shorter than six months, recovery is not taken into consideration [15]. Spinal bone metastases can also be considered for three courses of radiotherapy, if the cord dose can be limited to safe levels [13]. Eventually, prospective studies are needed to define the tolerance doses and suitable fractionation regimens.

## Conclusions

Re-irradiation including repeat re-irradiation has the potential to provide worthwhile symptom palliation and/or temporary tumor growth arrest, thereby contributing to the ever-increasing armamentarium of options that increase the survival of patients with incurable cancer and try to prolong the time period where independent living is possible.
